# Everybody's Page

**Published:** 1890-07-19

**Authors:** 


### EVERYBODY'S PAGE.

THE *New York Medical Journal*, on the subject of the report that American ladies take strychnine "Pick-me-up" lozenges, says that "American ladies will no doubt be interested in learning that they have this addition to their other picturesque habits credited to them by observant foreigners."

---

THERE is no period of life more important, from an hygienic point of view, than the age between 12 and 21, for as Dr. Hector George points out, it is between these years that the skeleton and brain take their definite form. It is then that an abundance of general exercise will facilitate development; it is during this period that deformities are so easily contracted; stooping, for instance, from leaning over work, or writing at a too low desk, crooked shoulders or hips from carrying weights always on one side, or from allowing pupils to sit crookedly at their work. But now, happily, there is open to everybody that splendid antidote to deformity—gymnastics. It is the same thing with the brain, everything in a man's life depends on the impressions he receives in his youth; his surroundings must be refined and clever if it is wished that his mind may also become so. In the words of Dr. George, what is wanted is "An upright mind in an upright body."

MOST men indulge in a cold bath every morning, knowing what a refreshing stimulating action the cold water has. Women, on the other hand, often use their baths much too hot, and stay in them far too long, and the effect is of necessity enervating. A bath "with the chill off" can be used by anybody, and the best way to get used to it is to decrease the temperature of the bath water daily.

---

SOMEHOW, in these days, we should hardly have expected to have heard the little moral lessons which have been drawn indirectly from Miss Fawcett's splendid achievements. We are told a woman must not only be learned—that is nothing, if she is not sweet, good, and domesticated as well. Exactly so; but the mere blue-stocking has been so well snubbed that we hear very little of her, and education is found to be a very much more comprehensive thing than a stock of narrow knowledge. Cannot a sweet girl graduate be as good a wife and a far nobler companion than the woman who is so taken up with household affairs that she finds no moment wherein to improve her mind. A true woman, really appreciative of good education, tries to excel in household management as much as she would in classics or mathematics.

